# A multi-component intervention (NEXpro) reduces neck pain: a randomized controlled trial among Swiss office workers

**DOI:** 10.5271/sjweh.4254

**Published:** 2026-01-01

**Authors:** Andrea Martina Aegerter, Venerina Johnston, Thomas Volken, Gisela Sjøgaard, Markus Josef Ernst, Hannu Luomajoki, Achim Elfering, Markus Melloh

**Affiliations:** 1 Zurich University of Applied Sciences, School of Health Sciences, Winterthur, Switzerland.; 2 University of Lucerne, University Research Centre Health and Society, Faculty of Health Sciences and Medicine, Lucerne, Switzerland.; 3 University of Southern Queensland, School of Health and Medical Sciences, Ipswich, Australia.; 4 University of Southern Queensland, Centre for Health Research, Ipswich, Australia.; 5 University of Southern Denmark, Department of Sports Science and Clinical Biomechanics, Odense, Denmark.; 6 University of Birmingham, School of Sport, Exercise & Rehabilitation Sciences, Centre of Precision Rehabilitation for Spinal Pain, Birmingham, UK.; 7 University of Bern, Institute of Psychology, Bern, Switzerland.

**Keywords:** adherence, efficiency, ergonomics, exercise, health promotion, occupational health, patient compliance, workplace

## Abstract

**Objective:**

This study aimed to investigate the effect of a 12-week multi-component intervention on neck pain among Swiss office workers.

**Methods:**

Between January 2020 and April 2021, we conducted a stepped-wedge cluster randomized controlled trial involving 120 office workers (18−65 years) without severe neck problems from two Swiss companies. Participants started in the control condition and sequentially transitioned to the intervention condition by their cluster. The 12-week intervention included neck exercises, health-promotion workshops, and workplace ergonomics. Neck pain was assessed by intensity [numeric rating scale (NRS) 0=no pain, 10=maximum pain], frequency (days with neck pain in the past 28 days), and disability [Neck Disability Index (NDI) 0%=no disability, 100%=maximum disability). Mixed-effects models were used to assess the intervention’s effect on neck pain intensity, frequency, and disability.

**Results:**

This analysis includes 517 observations (295 control, 222 intervention). At baseline, the mean age was 43.7 years [standard deviation years (SD) 9.8], and 71.7% were female. The average neck pain intensity was NRS 2.4 (SD 2.0), frequency 6.8 days (SD 8.0), and disability 11.8% (SD 9.9). A statistically significant effect favoring the multi-component intervention was found for neck pain frequency, with a marginal predicted mean reduction of 1.55 days [95% confidence interval (CI) -2.84−-0.26], and neck disability, with a marginal predicted mean reduction of NDI 2.23% (95% CI -2.96−-1.68).

**Conclusions:**

This study provides evidence of the effectiveness of a 12-week multi-component intervention in reducing neck pain among office workers. Specifically, office workers experienced neck pain less frequently and with a milder impact on daily activities. Further research is needed to investigate long-term effects.

Globally, neck pain is a prevalent condition associated with disability, reduced quality of life, and healthcare costs (1). Workers with neck pain manage their problems in various ways such as taking time away from work, submitting a claim for workers’ compensation, or remaining at work but performing sub-optimally, resulting in productivity loss and reduced quality of work (2, 3). One occupational group at greater risk of neck pain is office workers due to the static postures, adopted with an annual incidence of up to 63% per year (4, 5). Due to the multifactorial nature of neck pain, solutions need to consider not only the individual worker but also the physical and psychosocial environment that likely contributes to the onset and maintenance of symptoms (6).

Interventions have tested a range from singular changes to the work environment [eg, implementing armrests (7)], to complete assessment of the workstation, a variety of exercises such as stretching, and strengthening, and more holistic interventions like workplace health promotion initiatives (8). In one study, both exercise and ergonomic modifications significantly reduced the frequency of neck pain in office workers (9). Shariat et al (10) went a step further and randomly allocated office workers to receive one of three interventions for six months: exercise only, ergonomic intervention only, and exercise plus ergonomic modifications. In comparison to the control group, this trial found that neck pain severity significantly improved after 4 months in all groups with no difference between intervention groups. The authors concluded that long-term benefits for musculoskeletal pain should not rely solely on ergonomic modifications but include exercises as well. The preponderance of evidence from systematic reviews supports the inclusion of exercise with equivocal evidence for ergonomic interventions with their benefit likely to be enhanced in combination with other interventions (11, 12).

Another intervention increasingly being delivered in the workplace is wellness and health promotion programs to positively influence workers’ physical and psychological health (13). With evidence that high levels of physical activity may protect against neck pain (5), the need to promote health and well-being is a valuable addition to any workplace intervention. A recent comparative effectiveness study implemented a 12-week ergonomic and exercise training intervention and an ergonomic and health promotion intervention to alleviate neck pain among office workers (14). The former intervention was found to be more effective in reducing neck pain intensity among all workers immediately following the intervention, which was not maintained at 12 months. Thus, it is possible that an intervention that includes exercise, ergonomic interventions and health promotion is likely to deliver benefits for office workers with neck pain not only in the short- but also longer term.

In summary, existing literature suggests that effectively addressing neck pain among office workers requires a multi-component approach. While exercise, health promotion, and ergonomic interventions are among the most studied and promising strategies, their combined (ie, additive) effect has not yet been sufficiently investigated. Although our research group previously demonstrated that this combined approach reduces neck pain-related productivity loss and headache-related outcomes (15, 16), its direct effect on neck pain remains unclear. The present analysis, using data from the same trial, addresses this question and provides results that support and extend our earlier findings on productivity loss (15).

Therefore, the aim of this work was to determine the effect of a 12-week multi-component intervention, combining current evidence-based interventions of exercise, ergonomic modifications and health education for neck pain among Swiss office workers. To address the observed limitation in previous studies of low adherence, this study also included an app offering feedback on and reminders to exercise.

## Methods

### Study design

This work is part of the project “Neck Exercise for Productivity” (NEXpro). The primary outcome of the NEXpro trial was neck pain-related work productivity loss, which has been reported in a separate publication (15). The present paper focuses on the secondary outcome of the NEXpro trial: neck pain.

**Figure 1:**
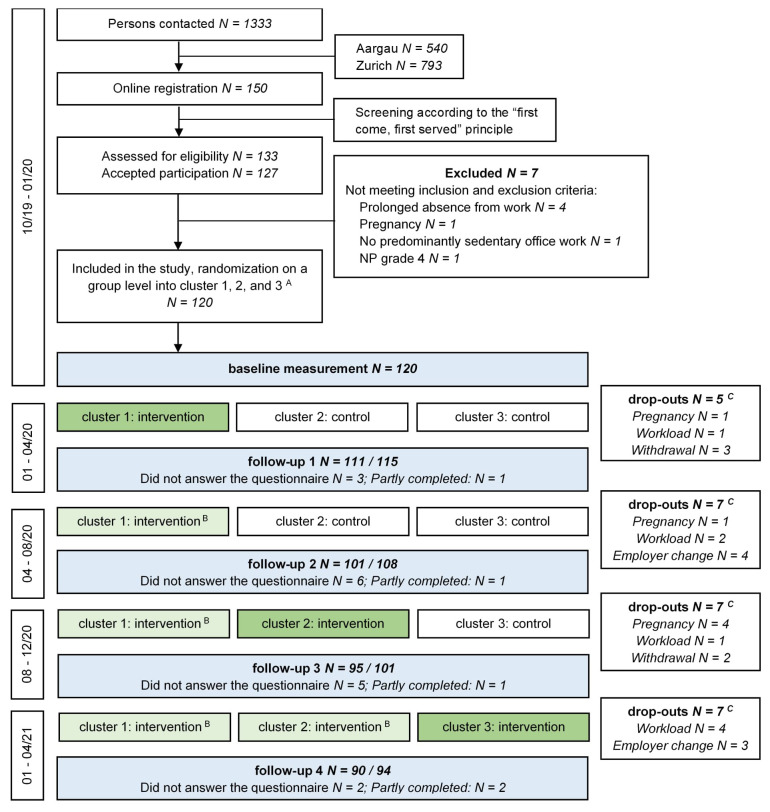
Trial flow chart.   A: Each cluster consists of five groups with eight participants each (N = 40). B: Unsupervised intervention.  C: N=107 participants started the intervention at the allocated time point, N=7 dropped out during the (supervised) intervention period (group 1: N=3; group 2, N=2; group 3, N=2), N=100 completed the (supervised) intervention, 94 completed the full trial (attrition rate of 22%).  Further comments: No intervention from 04–08/20 due to the COVID-19 pandemic. Participation rate Aargau: 10.4% (56 of 540 office workers) and Zurich 8.1% (64 of 793 office workers).

As part of the NEXpro project, we conducted a stepped-wedge cluster randomized controlled trial (RCT), a design that allows all participants to receive the intervention but at different time points (figure 1). Specifically, participants in cluster 1 received the intervention from January to April 2020. Cluster 2 served as a control group during the same period and received the intervention from May to August 2020. Cluster 3 remained in the control group throughout 2020 and received the intervention from January to April 2021 – four months later than originally scheduled (September to December 2020) due to the COVID-19 pandemic. Once participants transitioned from the control to the intervention condition, they remained in the intervention group for the remainder of the study. Detailed information on the study design and COVID-19-related adaptions is available in the study protocol (17) and the publication reporting our primary outcome (15).

The Ethics Committee of the Canton of Zurich, Switzerland approved the study (2019-01678). The CONSORT 2010 Statement extension to cluster randomized trials was used to guide the reporting of this RCT (18).

### Participants

*Recruitment.* Participants were recruited between October and December 2019 from two mid-sized companies located in the Swiss cantons of Aargau and Zurich. One company operated in the education sector, the other in the governmental sector. These companies were selected because they were the only ones that met the key requirement of allowing the intervention to be implemented during working hours. Moreover, their size justified the resource-intensive nature of the onsite intervention, including travel time for the research team, which would have been impractical in several smaller companies. Within each company, study information was distributed to all employees via email, the intranet, and presentations during lunch break meetings.

*Eligibility criteria.* Eligible participants were office workers aged 18–65 years who worked an average of >25 hours a week. Those with or without neck pain, able to speak and write German, and who had provided their consent were eligible (15, 17). Exclusion criteria were serious health problems according to the European Task Force recommendations (19), such as having suffered from neck trauma or injury previously (19) or being diagnosed with neck pathology. Furthermore, we excluded people who had undergone surgery on the neck previously (15, 17), had an upcoming absence from work for ≥4 weeks, were pregnant, or had any contraindications to perform the planned neck exercises in this study.

*Randomization.* Participants were first grouped into 15 subgroups, each consisting of eight individuals. To minimize intervention contamination, participants from the same company who worked in the same room or on the same floor were assigned to the same subgroup. These subgroups were then randomly allocated to one of three clusters (cluster 1, 2 or 3) by a senior biostatistician, who was blinded to participant identities. The timing of each cluster’s transition from the control to the intervention group was prespecified (see study design section). Accordingly, all participants within a given cluster switched to the intervention condition simultaneously based on their assigned schedule.

### Control

During the control period, participants received no intervention. They were asked to continue their usual activities until the intervention commenced.

### Intervention

The 12-week onsite multi-component intervention included an ergonomic intervention of the workplace, a health-promotion intervention seminar, and exercises of the neck (15, 17).

*Workplace ergonomics.* An evaluation of the participants’ workplace ergonomics was conducted at their office workstation, based on guidelines adapted to the Swiss context (20). Based on these assessments, adjustments were made to monitors, desks, and chairs. Importantly, these modifications were cost-neutral for the company as they involved optimizing existing resources and infrastructure.

*Health-promotion seminars*. Weekly 45-minute health-promotion seminars, covering a broad range of health topics, were organized in a group setting and conducted onsite at the companies’ facilities. Topics included the musculoskeletal system, goal setting, physical exercise, mental health, self-efficacy, occupational stress, ergonomics and digital media, conflict resolution, sleep and relaxation, healthy nutrition, mindfulness and resilience, and sustaining motivation. The content of the 12 seminars was informed by previous research (3), feedback from of the two participating companies, and input from experts at international academic partner institutions.

*Neck exercises.* In total, study participants were instructed to perform three neck exercise sessions per week, each lasting ≥20 minutes, amounting to one hour of neck exercises weekly (36 sessions over 12 weeks). Of these, one session per week was supervised and conducted onsite at the companies’ facilities in a group setting, while the remaining two sessions were self-administered.

Each participant received a set of 18 exercises targeting the upper body and neck (3, 15, 17). Detailed descriptions of all exercises are provided in the supplementary material (www.sjweh.fi/article/4254), figure S1). Each session began with warm-up exercises (eg, low-intensity exercises), followed by strengthening and concluded with cool-down exercises (eg, stretching exercises). The training load of the strengthening exercises was based on the participant’s 10-repetition maximum (10-RM), performed in 2–3 sets of 10−15 repetitions. Training intensity was assessed at weeks 3, 6, and 9. Rest intervals between sets were incorporated to prevent overexertion. Participants used an app (Physitrack®, London, UK), accessible via desktop, tablet, or smartphone, which provided training reminders, feedback, and monitored training adherence.

*Training and setting.* All interventions were delivered by movement scientists, physiotherapists, or psychologists, all of whom received ≥4 hours of training prior to the start of the intervention. Time spent participating in the intervention was counted as working time for all participants. Because each cluster contained a different number of subgroups from the two companies, the group sizes for the onsite training sessions (both seminars and neck exercises) varied accordingly. After completion of the supervised intervention period, the research team encouraged all participants to continue the exercises independently and without supervision.

*COVID-19 pandemic.* From March 2020 onwards, the intervention was delivered in a hybrid format due to the COVID-19 pandemic. Specifically, ergonomic assessments and intervention were conducted at the workplace where participants spent most of their time (eg, at home). During the assessments, emphasis was placed on empowering participants to independently apply ergonomic principles to other workplaces, a practice that had been part of the intervention even before the pandemic. Neck exercises and workshops continued to be conducted in a group setting, delivered digitally via Zoom or Microsoft Teams.

Office workers’ access to the workplace during the pandemic was subject to company-specific regulations (eg, ≤3 people per room) and national Swiss guidelines.

### Outcomes

Data were collected from all participants at five time points: baseline, 4, 8, 12, and 16 months. Data were stored using the tool UNIPARK© (Berlin, Germany) and collected via online questionnaires. Participants were allowed to report their time spent completing the questionnaires as working time.

*Neck pain.* Neck pain was assessed at all five time points across three dimensions: intensity, frequency, and disability. Intensity was measured as the average neck pain during the past four weeks using a numerical rating scale (NRS) ranging from 0 (no pain) to 10 (maximum pain). The NRS demonstrates high test-retest reliability and validity in adult pain assessment (21). Frequency was defined as the number of days participants experienced neck pain within the past four weeks (0–28 days). Disability was assessed using the German version of the Neck Disability Index (NDI), scored from 0% (no disability) to 100% (maximum disability). The NDI is the most widely used and validated tool for clinical self-assessment and research in neck pain (22) The German NDI version reports a minimal detectable change of 7 points on the original 0–50-point scale in neck pain patients, corresponding to 14% on the 0–100% scale (22).

*Covariates.* The following information was collected from participants only at baseline: age, gender (male, female), nationality (Swiss, non-Swiss), and employer (Zurich, Aargau). Information expected to change over time were collected at all five time points: civil status (married, not married but in a relationship, not married and not in a relationship), education level (tertiary level education, non-tertiary level education), average monthly earnings (in Swiss Francs, CHF), workload percentage (<80, 80–89, 90–99, 100%), and work role (with or without leadership responsibilities).

Work-related stress was assessed using the Job-Stress-Index (JSI), which reflects the ratio of work-related resources to stressors. The index consists of 38 validated items distributed across 10 subscales (23), with 6 subscales assessing stressors (uncertainty, work organization issues, time pressure, qualitative overload, social stressors from supervisors and colleagues), and 4 subscales capturing resources (scope for action, holistic approach to work, general appreciation, and supportive supervisor behavior). The JSI is scored 0–100, with interpretation recommended by the scale authors as follows: scores <45.880 indicate more resources than stressors; scores of 45.880–54.122 reflect a balanced ratio; and scores >54.122 indicate more stressors than resources (23).

Adherence to the intervention was defined as the proportion of sessions attended relative to both the total and the recommended number of sessions.

Additionally, physical measurements, including neck muscle strength assessments, were conducted at each measurement time point (24). However, these data are only relevant within the context of safety events for the present paper.

### Sample size calculation

The sample size calculation was based on the primary outcome of neck pain-related work productivity loss (15, 17). A baseline work productivity of 90% and an intervention-related work productivity increase of 5% were assumed for sample size calculation (3). Alpha was set at 0.05 (type I error), and beta at 20% (power =80%, type II error). Due to the attrition rate of nearly 20% in a previous Australian study by Pereira et al (3), we increased the number of groups and subjects per group by 20% each. Thus, we enrolled 120 participants over 15 groups for four measurement time points (480 observations). An additional measurement time point was added (follow-up 4) due to the COVID-19 pandemic, increasing the number of measurement time points to 5 and the number of observations to 600.

### Statistical analysis

*Descriptive statistics.* To characterize participants, mean, median, standard deviation (SD), maximum, and minimum values were used. Relative and absolute frequencies were reported, where variables were nominal or ordinal.

*Mixed-effects models.* Three mixed-effects models were fitted to the data to estimate the change in neck pain, with neck pain intensity, frequency, and disability as dependent variables. Each model included random intercepts for participants (participant ID) and clusters (cluster 1, 2 and 3), as well as fixed effects for time (baseline, 4, 8, 12, and 16 months) and treatment group (intervention, control) following the standard parametrization of stepped wedge cluster randomized models and the study protocol (17). In addition to the unadjusted models, adjusted models were calculated to control for covariates. Following the primary outcome paper and our statistical analysis plan (15, 17), we adjusted for age, gender, education, civil status, nationality, employer, workload percentage, work role, and work-related stress conditions (JSI) in all three models. Statistical significance was set at P<0.05.

*Intensity.* We employed mixed-effects models of the beta family to estimate unadjusted and adjusted mean neck pain intensity. To meet the requirements of the beta regression model, the original neck pain intensity scale was rescaled to a range of 0–1. Regression coefficients with corresponding 95% confidence intervals (CI) represent transformed scores on the logit scale. We reported all marginal effects on the original neck pain intensity scale with a corresponding 95% CI.

*Disability.* We employed mixed-effects models of the beta family to estimate unadjusted and adjusted neck pain disability. To meet the requirements of the beta regression model, the original NDI scale was rescaled to a range of 0–1. Regression coefficients with corresponding 95% CI represent transformed scores on the logit scale. We reported all marginal effects on the original NDI scale with a corresponding 95% CI.

*Frequency.* We employed mixed-effects models of the zero-inflated negative binomial family with log links to estimate unadjusted and adjusted neck pain frequency.

### Overall procedures

Statistical analyses were performed using R version 3.6.3. Analysts were blinded to the identity of participants. Data were analyzed on an intention-to-treat basis (17). The scientific validity, integrity, and safety of this trial were assessed independently through a data monitoring committee.

## Results

### Participant flow

Of the 1333 individuals who were contacted during recruitment between 28 October 2019 and 20 December 2019, 150 expressed interest in participating in the study via the online registration platform (figure 1). On a first-come, first-served basis, 133 of these individuals were assessed for eligibility. Of those, 6 declined to participate, and an additional 7 were excluded for not meeting the inclusion or exclusion criteria, resulting in a final sample of 120 participants. All 120 participants initiated the study and completed the baseline measurement; however, 26 participants dropped out either before (N=13), during (N=7), or after (N=6) their assigned intervention period (male: 9; female: 17), corresponding to an attrition rate of 22%. This resulted in a total of 517 observations (295 control, 222 intervention) out of a theoretical maximum of 600 (see supplementary material, table S4), with an average of 4.3 observations per participant, which were included in the mixed-effects models.

### Participants’ characteristics at baseline

Participants’ characteristics at baseline (N=120), including neck pain, workload percentage, and work-related stress conditions, are presented in table 1. At baseline, the mean age was 43.7 (SD 9.8) years, the majority were female (N=86, 71.7%), Swiss (N=95, 79.2%), in a relationship (married: N=48, 40%; not married: N=53, 44.2%), and had a tertiary level education (N=89, 74.2%) with a balanced distribution by employer (Zurich: 53.3%, N=64). Most participants worked full-time (N=67, 55.8%), had no leadership responsibilities (N=76, 63.3%), with an average monthly income of CHF 7679 (SD 2818). The mean neck pain intensity was 2.4/10 on the NRS (SD 2.0), with a neck pain frequency of 6.8 days out of 28 (SD 8.0), and a neck disability index of 11.8% (SD 9.9). Overall, 88% of participants (N=106) experienced neck pain at least once during the measurement periods. Among the 79.2% of participants (N=95) who reported neck pain at baseline, the mean pain intensity was at NRS 3.0 (SD 1.8, median 2.0, minimum 0.0, maximum 9.0, interquartile range 2.0).

**Table 1:** Participant characteristics at baseline (N=120) [NDI=Neck Disability Index, NRS=Numeric Rating Scale, SD=standard deviation.]

		Mean (SD)	N (%)
Neck pain intensity [NRS 0=no pain to 10=maximum pain]		2.4 (2.0)	
Neck pain frequency [number of days with neck pain within last 4 weeks]		6.8 (8.0)	
Neck disability [NDI, 0 to 100%]		11.8 (9.9)	
Workload percentage			
	< 80		25 (20.8)
	80–89		28 (23.3)
	90–99		19 (15.8)
	100		48 (40.0)
Job-Stress-Index [0-100]		47.6 (5.0)	
Job-Stress-Index [categories]			
	Favourable range (JSI below 45.879; resources > stressors)		50 (41.7)
	Sensitive range (JSI between 45.880 and 54.122; resources = stressors)		54 (45.0)
	Critical range (JSI above 54.123; resources < stressors)		16 (13.3)

### Adherence to intervention

Adherence to the interventions is presented in table 2. Data from 107 participants were included in this analysis (120 minus the 13 who dropped out prior to starting the intervention). Of these 107 participants, 27% (N=29) were classified as adherent to neck exercises (mean=31.2, range=0–83 sessions), 61.7% (N=66) adherent to the health-promotion information (mean=8.2, range= 0–12 group workshop attendances), and 97.2% (N=104) adherent to workplace ergonomics recommendations.

**Table 2:** Adherence to intervention (N=107)^a^. [IQR=interquartile range; SD=standard deviation.]

				Mean (SD)	Median (IQR)	N (%)
Number of neck exercise training sessions over 12 weeks				31.2 (13.3)	31.0 (10.5)	
Adherence to neck exercises ^b^						
	Adherent					29 (27.1)
		Exceeded (>3 sessions/week)				25 (23.4)
		Met (3 sessions/week)				4 (3.7)
	Non-adherent					78 (72.9)
		Slightly below (2–<3 sessions/week)				56 (52.3)
		Clearly below (1–<2 sessions/week)				13 (12.2)
		Very clearly below (<1 session/week)				9 (8.4)
Health-promotion information group workshops attended over 12 weeks				8.2 (2.8)	8.0 (3.0)	
Adherence to workshops ^c^						
	Adherent					66 (61.7)
		Exceeded (>8 workshops attended)				52 (48.6)
		Met (8 workshops attended)				14 (13.1)
	Non-adherent					41 (38.3)
		Slightly below (5–7 workshops attended)				32 (29.9)
		Clearly below (<5 workshops attended)				9 (8.4)
Adherence to workplace ergonomics						
	Adherent					104 (97.2)
	Non-adherent					3 (2.8)

^a^ Of 120 participants, 107 started their assigned intervention period (N=13 dropouts prior to the intervention). ^b^ Participants followed our recommendation to exercise 3 times a week over 12 weeks (≥36 sessions=adherent, <36 sessions=non-adherent). ^c^ Participants followed our recommendation to attend a minimum of 8–12 workshops (≥8=adherent, <8=non-adherent). Due to the COVID pandemic, 4 health-promotion information group workshops could not be held, and materials were sent to participants to be completed independently.

### Mixed-effects models

For all mixed-effects models, both unadjusted and adjusted analyses were performed using a total of 517 observations. Adjusted models controlled for age, gender, education, civil status, nationality, employer, workload percentage, work role, and work-related stress conditions. All adjusted models are presented in tables 3–5, while unadjusted models can be found in the supplementary tables S1–3). Additionally, to facilitate interpretation, table 6 provides an overview of the marginal effects along with a direct comparison between the adjusted and unadjusted models.

**Table 3:** Neck pain intensity ^a^ adjusted model. [CI=confidence interval].

		Coefficient	95% CI	P-value
Intercept		-1.50	-2.18−-0.81	<0.0001
Treatment, intervention (Ref = control)		-0.25	-0.57−0.06	0.12
Measurement time point (Ref = Baseline January 2020)				
	Follow-up 1 (April 2020)	-0.26	-0.55−0.03	0.07
	Follow-up 2 (August 2020)	-0.14	-0.43−0.15	0.35
	Follow-up 3 (November 2020)	-0.02	-0.37−0.32	0.91
	Follow-up 4 (April 2021)	-0.34	-0.76−0.09	0.12
Age		-0.03	-0.05−-0.005	0.02
Gender, male (Ref = female)		-0.09	-0.58−0.41	0.73
Education, tertiary (Ref = non-tertiary level)		-0.62	-1.16−-0.07	0.03
Civil status (Ref = married)				
	Not married, in a relationship	0.39	-0.09−0.87	0.12
	Not married, not in a relationship	0.51	-0.13−1.14	0.12
Nationality, non-Swiss (Ref = Swiss)		0.35	-0.17−0.88	0.19
Employer, Aargau (Ref = Zurich)		-0.11	-0.55−0.33	0.62
Workload percentage (Ref = 100%)				
	90−99%	-0.28	-0.88−0.32	0.36
	80−89%	0.04	-0.48−0.56	0.89
	<80%	0.54	-0.07−1.15	0.08
Work role, with leadership responsibilities (Ref: without leadership responsibilities)		0.35	-0.14−0.84	0.17
Job Stress Index		0.02	-0.01−0.05	0.16

^a^ Neck pain intensity was measured using the Numeric Rating Scale (NRS) from 0–10. Parameter estimates are based on the logit model scale of transformed neck pain intensity scores. Marginal effects are provided to assist in the interpretation of these results and are shown in table 6.

**Table 4:** Neck disability ^a^ adjusted model. [CI=confidence interval]

		Coefficient	95% CI	P-value
Intercept		-2.53	-3.16−-1.90	<0.0001
Treatment, intervention (Ref = control)		-0.35	-0.63−-0.07	0.02
Measurement time point (Ref = Baseline, January 2020)				
	Follow-up 1 (April 2020)	-0.13	-0.38−0.13	0.33
	Follow-up 2 (August 2020)	-0.13	-0.39−0.13	0.33
	Follow-up 3 (November 2020)	-0.03	-0.34−0.28	0.85
	Follow-up 4 (April 2021)	-0.18	-0.56−0.21	0.37
Age		-0.02	-0.04−-0.0002	0.048
Gender, male (Ref = female)		-0.004	-0.45−0.44	0.99
Education, tertiary (Ref = non-tertiary level)		-0.44	-0.94−0.06	0.09
Civil Status (Ref = married)				
	Not married, in a relationship	0.42	-0.02−0.86	0.06
	Not married, not in a relationship	0.31	-0.27−0.90	0.29
Nationality, non-Swiss (Ref = Swiss)		0.27	-0.21−0.74	0.28
Employer, Aargau (Ref = Zurich)		-0.12	-0.52−0.29	0.57
Workload percentage (Ref = 100%)				
	90−99%	0.04	-0.51−0.59	0.89
	80−89%	0.05	-0.43−0.53	0.84
	< 80%	0.53	-0.03−1.09	0.06
Work role, with leadership responsibilities (Ref: without leadership responsibilities)		0.37	-0.08−0.81	0.11
Job Stress Index		0.03	0.01−0.06	0.02

^a^ Neck disability was measured using the Neck Disability Index (NDI) from 0 to 100%. Parameter estimates are based on the logit model scale of transformed neck disability index scores. Marginal effects are provided to assist in the interpretation of these results and are shown in Table 6

**Table 5:** Neck pain frequency ^a^ adjusted model. [CI=confidence interval].

		Coefficient	95% CI	P-value
Intercept		1.40	0.72−2.07	<0.0001
Treatment, intervention (Ref = control)		-0.35	-0.61−-0.10	0.007
Measurement time point (Ref = Baseline, January 2020)				
	Follow-up 1 (April 2020)	0.001	-0.22−0.22	0.99
	Follow-up 2 (August 2020)	0.01	-0.22−0.24	0.94
	Follow-up 3 (November 2020)	0.21	-0.06−0.48	0.13
	Follow-up 4 (April 2021)	0.22	-0.13−0.57	0.22
Age		-0.003	-0.03−0.02	0.80
Gender, male (Ref = female)		-0.12	-0.63−0.38	0.63
Education, tertiary (Ref = non-tertiary level)		-0.15	-0.70−0.40	0.59
Civil status (Ref = married)				
	Not married, in a relationship	0.03	-0.46−0.53	0.90
	Not married, not in a relationship	0.29	-0.36−0.94	0.39
Nationality, non-Swiss (Ref = Swiss)		0.04	-0.49−0.57	0.87
Employer, Aargau (Ref = Zurich)		-0.31	-0.77−0.14	0.17
Workload percentage (Ref = 100%)				
	90−99%	0.52	-0.09−1.13	0.09
	80−89%	0.26	-0.27−0.79	0.34
	<80%	0.56	-0.05−1.17	0.07
Work role, with leadership responsibilities (Ref: without leadership responsibilities)		0.36	-0.13−0.86	0.15
Job Stress Index		0.004	-0.02−0.03	0.80

^a^ Neck pain frequency was quantified by the number of days with neck pain within the past 28 days. Parameter estimates are based on the model scale of transformed days with neck pain. Marginal effects are provided to assist in the interpretation of these results and are shown in Table 6.

**Table 6:** Overview of marginal effects ^a^. [CI=confidence interval; NDI=Neck Disability Index; NRS=Numeric Rating Scale].

			Coefficient	95% CI
Neck pain intensity (NRS, 0−10)				
	Adjusted model			
		Intervention group	1.31	0.53−2.91
		Control group	1.63	0.72−3.30
		Predicted difference between groups	-0.32	-0.43−-0.23
	Unadjusted model			
		Intervention group	1.33	0.90−1.93
		Control group	1.61	1.24−2.06
		Predicted difference between groups	-0.28	-0.39−-0.21
Neck disability (NDI, 0−100%)				
	Adjusted model			
		Intervention group	5.77	2.41−13.19
		Control group	8.00	3.61−16.77
		Predicted difference between groups	-2.23	-2.96−-1.68
	Unadjusted model			
		Intervention group	5.82	3.96−8.47
		Control group	7.94	6.15−10.20
		Predicted difference between groups	-2.13	-2.83−-1.60
Neck pain frequency (days with neck pain)				
	Adjusted model			
		Intervention group	3.69	1.39−9.75
		Control group	5.24	2.10−13.06
		Predicted difference between groups	-1.55	-2.84−-0.26
	Unadjusted model			
		Intervention group	3.69	2.50−5.44
		Control group	5.18	3.96−6.77
		Predicted difference between groups	-1.49	-2.78−-0.20

^a^ Marginal effects are presented to facilitate interpretation, with predicted values for the control and intervention groups, as well as the predicted differences between groups. Both adjusted and unadjusted models are reported for direct comparison. Adjusted models control for age, gender, education, civil status, nationality, employer, workload percentage, work role, and work-related stress conditions.

*Intensity.* Adjusted for all covariates, no association was found for the intervention with neck pain intensity (-0.25; 95% CI -0.57−0.06; table 3). Specifically, the predicted NRS values were 1.63 (95% CI 0.72−3.30) in the control group and 1.31 (95% CI 0.53−2.91) in the intervention group (table 6). The predicted difference in mean NRS between groups was -0.32 (95% CI -0.43−-0.23).

*Disability.* Adjusted for all covariates, the intervention was negatively associated with neck disability (-0.35; 95% CI -0.63−0.07; table 4). Specifically, the predicted NDI values were 8.00 (95% CI 3.61−16.77) in the control group and 5.77 (95% CI 2.41−13.19) in the intervention group (table 6). The predicted difference in mean NDI between groups was -2.23 (95% CI -2.96−-1.68), with the negative sign indicating an intervention-related reduction in the NDI. Furthermore, higher neck disability was associated with younger age (-0.02, 95% CI -0.56−-0.0002) and increased work-related stress conditions (0.03; 95% CI 0.01−0.06). No association was found for measurement time points, the two different organizations, gender, education level, civil status, nationality, workload percentage, and work role with neck disability.

*Frequency.* Adjusted for all covariates, the intervention was negatively associated with neck pain frequency (-0.35; 95% CI -0.61−-0.10; table 5). Specifically, the predicted values were 5.24 days with neck pain (95% CI 2.10−13.06) in the control group and 3.69 days with neck pain (95% CI 1.39−9.75) in the intervention group (table 6). The predicted difference in mean numbers of days with neck pain between groups was -1.55 days (95% CI -2.84−-0.26), with the negative sign indicating an intervention-related reduction in the frequency of neck pain. Furthermore, no association was found for measurement time points, the two different organizations, age, gender, education, civil status, nationality, workload percentage, work role, and work-related stress conditions with neck pain frequency.

### Safety

One adverse event occurred following the physical examination of the neck (ie, hearing loss and tinnitus), with the participant requiring medical consultation.

## Discussion

This study aimed to evaluate the effect of a 12-week multi-component intervention, which included workplace-based exercise, ergonomic modifications, and health education, on neck pain outcomes among Swiss office workers. Of the three neck pain outcomes recorded in this study, a statistically significant effect was found for neck pain frequency and neck disability, with no significant effect observed for neck pain intensity. In essence, office workers participating in this combined intervention could expect to experience neck pain less frequently, and with less impact on daily activities when it occurs.

### Neck pain frequency

The frequency of neck pain in this study was significantly reduced by 1.55 days over a 28-day period with the multi-component intervention. Specifically, participants in the control group reported an average of 5.24 days with neck pain compared to 3.69 days in the intervention group. Compared to the baseline mean value of 6.8 days, the intervention group experienced a relative reduction of nearly 45%. Applying this relative reduction to a large epidemiological study of >6000 working-age adults, where 44% reported ≥1 day of neck pain in the past 7 days (25), and extrapolating this to a 28-day recall period (approximately 4 days), a similar relative reduction would correspond to about 2 fewer days with neck pain. In a sample of 6000 individuals, this would amount to approximately 5280 fewer days with neck pain over 28 days, or, viewed differently, >14 years without neck pain for an individual.

Moreover, our findings align with those of Mehrparvar et al (9), who reported that 70% of office workers experienced neck pain in the month before a workplace intervention, which was reduced to approximately 45% after either an ergonomic or exercise intervention, representing a relative reduction in neck pain frequency of nearly 35%. This highlights the potential impact of our intervention, especially considering that >60% of workers experience a recurrent episode of neck pain within one year of their initial episode (26), making strategies to reduce the burden of this condition critical.

Besides these two comparisons, it is difficult to directly compare our findings with previous research due to differences in how neck pain frequency is measured. Some studies use categorical outcomes such as “once only,” “more than once but not every day,” and “every day” (9), while others focus on activity-limiting pain over the past month (5), or report frequency in weekly, monthly, or three-month intervals (27).

### Neck disability

The reduction in neck pain frequency observed in this study was accompanied by a small improvement in disability, with a decrease of 2.23% on the NDI. Specifically, participants in the control group reported an average of NDI 8.00%, whereas those in the intervention group reported NDI 5.77%. Compared to the baseline mean value of NDI 11.80%, the intervention group experienced a relative reduction of nearly 50%, which is comparable to the relative reduction seen in neck pain frequency. While the 2.23% decrease was statistically significant, it is unlikely to be clinically meaningful. The German version of the NDI reports a minimal detectable change of 14% for people with neck pain (22), which already exceeds our baseline mean, whereas a recent systematic review reported the smallest detectable change to be around 10% for uncomplicated neck pain (28). We anticipate that greater and clinically meaningful improvements in disability may be achievable in populations with more severe pain at baseline. Therefore, we would recommend adjusting the inclusion criteria accordingly in future studies.

### Neck pain intensity

While nearly 80% of participants reported neck pain at baseline, the mean baseline neck pain intensity was NRS 2.4. This relatively low level is not surprising, given that the cohort included individuals both with and without neck pain. Nevertheless, it is consistent with findings from other studies involving office workers. For example, Johnston et al (14) reported a mean baseline neck pain intensity of 1.5 on a 0–9 NRS. Notably, that study observed a 8% reduction in pain intensity following a similar exercise intervention, and a 3% reduction after a comparable health education program after 12 months (14). In our study, participants in the control group reported an average of NRS 1.63, whereas those in the intervention group reported NRS 1.31. Compared to the baseline mean value of NRS 2.4, the intervention group experienced a relative reduction of nearly 45%, which is comparable to the relative reduction seen in neck pain frequency and disability.

We did not attempt to categorize participants in this study as neck cases due to the stepped-wedge design and the recurrent, episodic nature of neck pain (27). However, such a multi-component intervention would likely have been more successful for those with greater neck pain at baseline (29, 30). Therefore, future studies may consider screening office workers to include those with current neck pain greater than, for example, NRS 3.0, as well as those at risk of developing neck pain, and provide follow-up support to ensure continued adherence.

### Adherence

A feature of this study was the use of an app to improve adherence to the exercise intervention. In addition to one supervised session per week, participants were encouraged to access an app that demonstrated each exercise, provided training reminders and feedback, and recorded exercise load and adherence (ie, PhysiApp®). We believe this approach was successful in increasing adherence, as 23.4% of participants exceeded the recommended dose of three exercise sessions per week for 12 weeks. While the app is unlikely to be the sole contributor to this level of participation, it certainly contributed to a higher rate compared to previous studies. Using the definition of ‘*percentage of all 36 exercise sessions offered’* to enable comparison with Pereira et al (3), adherence in the current study was 86.7%, while it was only 54% in the latter study, which tested a similar exercise and ergonomic intervention for 12 weeks. Future research should consider using strategies to enhance adherence to exercise and include a range of adherence measures to enable meaningful comparison with previous studies.

### Strengths and limitations

The key strength of this study is its stepped-wedge design, which meant that participants acted as their own controls, reducing variability that arises when comparing two different samples, and ensuring that all received the intervention. This design was especially advantageous given the COVID-19 challenges and was the only feasible way to deliver the onsite intervention with limited personnel resources and budget. A second strength was the low attrition rate of 22% over the course of the study. Loss to follow-up after 12-month workplace interventions is a common challenge for most researchers, with rates typically in the range of 45–65% (14, 29).

We would like to highlight four limitations. First, while the study design followed the criteria for randomized controlled trials, the impact of the COVID-19 pandemic on the results, particularly on the adherence to an onsite intervention, cannot be ruled out. Second, the sample size was determined based on the primary outcome of neck pain-related health productivity loss and may have been too small to detect changes in pain (eg, neck pain intensity). Third, the lack of change in neck pain intensity could also be due to the low baseline pain levels, which limited the potential for detecting significant improvements. Though participants with neck pain at baseline had only slightly higher average pain intensity than the entire sample, a subgroup analysis based on neck pain status at baseline was avoided because none of the assessments used to measure neck pain were suitable for reliable categorization (eg, due to their recall periods). Fourth, while the generalizability of our findings applies to our sample, it is limited to the broader population of office workers, especially considering that our participants were highly educated. Despite these limitations, we believe the results are useful to organizations wanting to offer all workers a low-cost intervention to improve neck pain. Information on cost-utility and cost-benefit analyses can be found in a separate paper (31).

### Concluding remarks

A 12-week workplace-based multi-component intervention, including evidence-based ergonomics, exercise, and health-promotion workshops, was effective in reducing the frequency of neck pain among office workers. There was a corresponding, albeit small, reduction in the degree of disability associated with this decrease in neck pain frequency. Further research should consider targeting such interventions to individuals at greater risk of neck pain to assess long-term effects.

## Supplementary material

Supplementary material

## Data Availability

The datasets generated and analyzed during the current study are available from the corresponding author on reasonable request.

## Protection of research participants

This study was performed in line with the principles of the Declaration of Helsinki. The Ethics Committee of the Canton of Zurich, Switzerland, provided approval (15.10.2019, swissethics no. 2019-01678).

## Trial registration

ClinicalTrials.gov, NCT04169646. Registered 15 November 2019. https://clinicaltrials.gov/ct2/show/NCT04169646.

## Study protocol

Aegerter AM, Deforth M, Johnston V, Ernst MJ, Volken T, Luomajoki H, et al. Onsite multi-component intervention to improve productivity and reduce the economic and personal burden of neck pain in Swiss office-workers (NEXpro): protocol for a cluster-randomized controlled trial. *BMC musculoskeletal disorders*. 2020;21 (1):391.
