# Supplementary material for: A multi-component intervention (NEXpro) reduces neck pain: a randomized controlled trial among Swiss office workers
Source: Scand J Work Environ Health. 2025 Dec 27;52(1):51–62. doi: 10.5271/sjweh.4254 (PMC12781098; doi:10.5271/sjweh.4254)
Supplement: Supplementary material [file SJWEH-52-51-S001.pdf]

A multi-component intervention (NEXpro) reduces neck pain: a randomized controlled trial among Swiss office workers[1]

by Andrea Martina Aegerter, PhD,[2] Venerina Johnston, PhD, Thomas Volken, PhD, Gisela Sjøgaard, PhD, Markus Josef Ernst, MSc, Hannu Luomajoki, PhD, Achim Elfering, PhD, Markus Melloh, PhD, on behalf of the NEXpro collaboration group

1. Supplementary material
2. Correspondence to: Andrea Martina Aegerter, Zurich University of Applied Sciences, School of Health Sciences, Katharina Sulzer-Platz 9, 8400 Winterthur, Switzerland. [E-mail: andrea.aegerter@unilu.ch] ORCID: 0000-0003-0249-6399

Table S1: Neck pain intensity, unadjusted model

	**Coefficient**	**95% confidence interval**	**p-value**
**Intercept**	-1.49	-1.75–-1.22	< 0.0001
**Treatment, intervention** (Ref = control)	-0.23	-0.54–0.09	0.16
**Measurement time point** (Ref = Baseline, January 2020)			
Follow-up 1 (April 2020)	-0.26	-0.55–0.02	0.07
Follow-up 2 (August 2020)	-0.17	-0.46–0.13	0.27
Follow-up 3 (November 2020)	-0.04	-0.38–0.31	0.83
Follow-up 4 (April 2021)	-0.38	-0.81–0.05	0.08

Key: Neck pain intensity was measured using the Numeric Rating Scale (NRS) from 0 to 10. Parameter estimates are based on the logit model scale of transformed neck pain intensity scores. Marginal effects are provided to assist in the interpretation of these results and are shown in Table 6.

Table S2: Neck disability, unadjusted model

	Coefficient	95% confidence interval	p-value
**Intercept**	-2.35	-2.59−-2.10	< 0.0001
**Treatment, intervention** (Ref = control)	-0.33	-0.62−-0.05	0.02
**Measurement time point** (Ref = Baseline, January 2020)			
Follow-up 1 (April 2020)	-0.13	-0.39−0.12	0.31
Follow-up 2 (August 2020)	-0.16	-0.42−0.11	0.24
Follow-up 3 (November 2020)	-0.04	-0.35−0.27	0.81
Follow-up 4 (April 2021)	-0.20	-0.59−0.18	0.31

Key: Neck disability was measured using the Neck Disability Index (NDI) from 0 to 100%. Parameter estimates are based on the logit model scale of transformed neck disability index scores. Marginal effects are provided to assist in the interpretation of these results and are shown in Table 6.

Table S3: Neck pain frequency, unadjusted model

	Coefficient	95% confidence interval	p-value
**Intercept**	1.57	1.33−1.82	< 0.0001
**Treatment, intervention** (Ref = control)	-0.34	-0.59−-0.09	0.01
**Measurement time point** (Ref = Baseline, January 2020)			
Follow-up 1 (April 2020)	-0.002	-0.23−0.22	0.98
Follow-up 2 (August 2020)	0.01	-0.22−0.24	0.92
Follow-up 3 (November 2020)	0.19	-0.08−0.46	0.16
Follow-up 4 (April 2021)	0.20	-0.14−0.55	0.25

Key: Neck pain frequency was quantified by the number of days with neck pain within the past 28 days. Parameter estimates are based on the model scale of transformed days with neck pain. Marginal effects are provided to assist in the interpretation of these results and are shown in Table 6.

Table S4: Number of observations

	N (N missing or dropout)	
**Total number of observations**	517 (83)	
	Control period	Intervention period
**Number of observations split by group**	295 (25)	222 (58)
Baseline	120 (0)	0 (0)
Follow-up 1	75 (5)	36 (4)
Follow-up 2	69 (11)	32 (8)
Follow-up 3	31 (9)	64 (16)
Follow-up 4	0 (0)	90 (30)

Figure S1: Set of neck exercises

**Bilateral scapular raise**	**Bilateral shoulder shrugs**	**Row**
3x10 repetitions	3x10 repetitions	3x10 repetitions
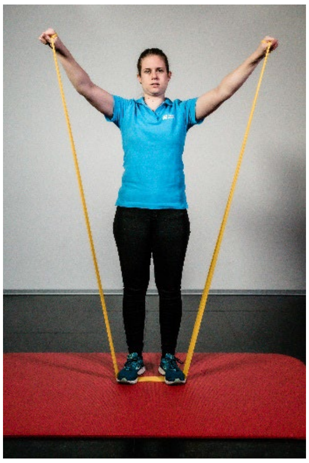	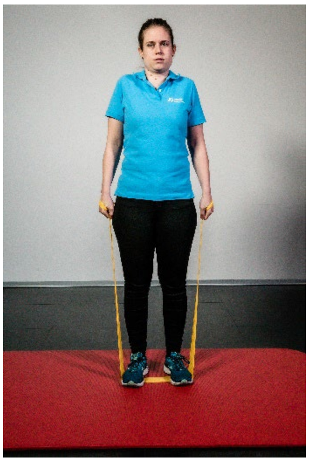	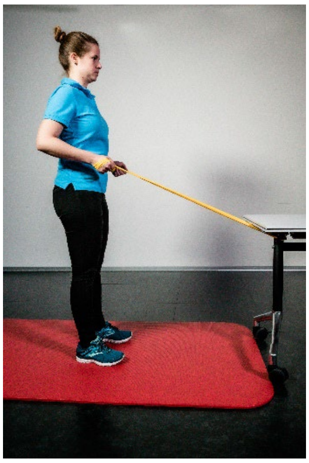
**Bilateral shoulder extension**	**Bilateral shoulder external rotation**	**Bench dips**
3x10 repetitions	3x10 repetitions	3x10 repetitions
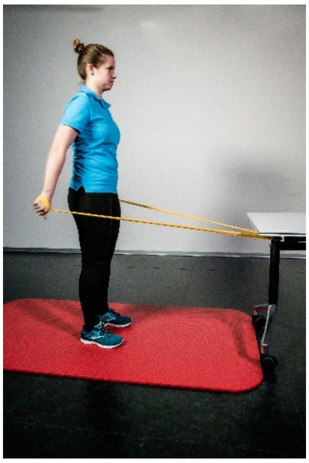	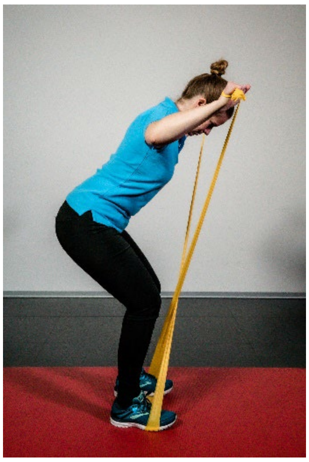	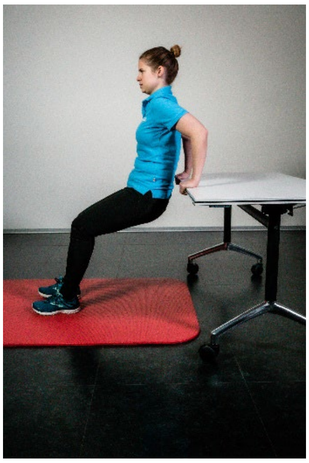

Seated side stretch

3x20 seconds, each side

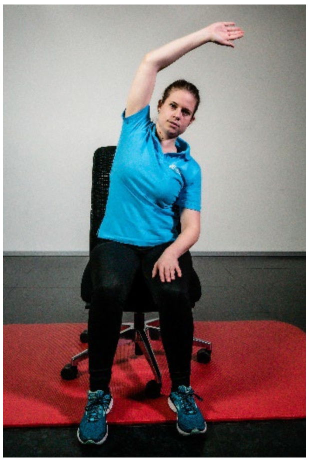

Self-massage

3x20 seconds

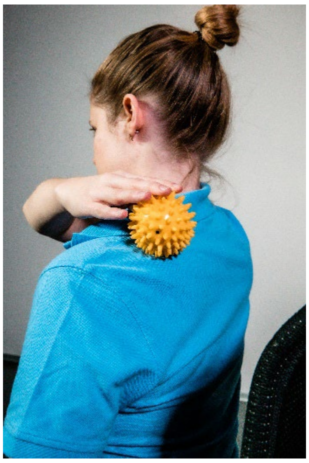

Isometric neck flexion

5x5 seconds

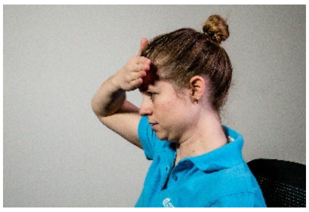

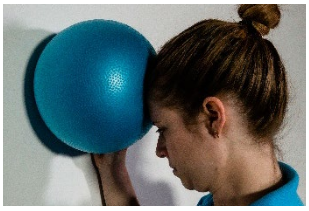

Isometric neck extension

5x5 seconds

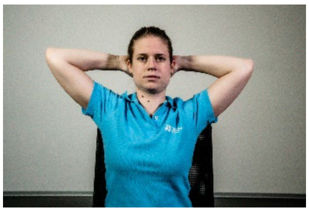

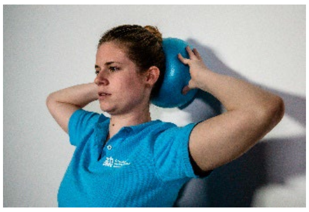

Isometric neck rotation

5x5 seconds

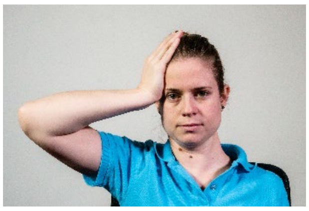

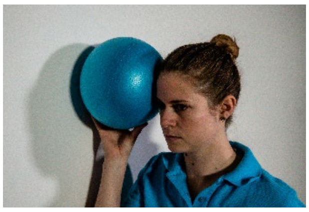

Push-ups

3x10 repetitions

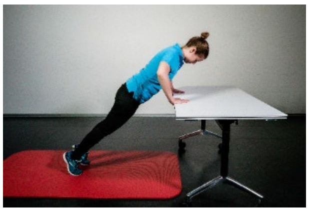

Bilateral shoulder circling

1x20 seconds

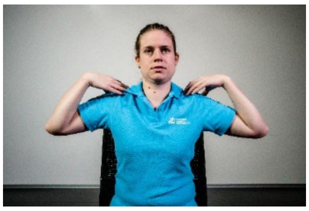

Upper body rotation

1x20 seconds

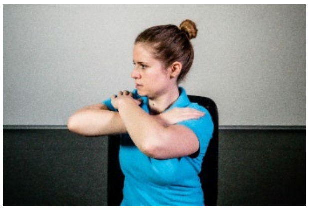

**Stretch of neck extensor**	**Lateral neck stretch**
3x20 seconds	3x20 seconds, each side
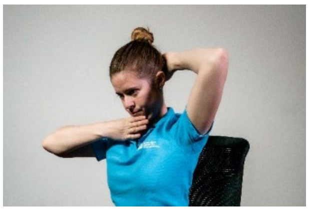	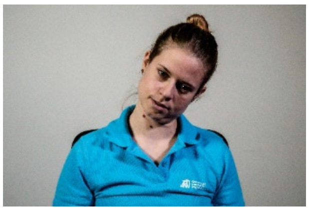
